# The Acute Effects of Ball Pressure on Anticipation Timing Following a Series of Purposeful Headers in Adult Football (Soccer) Players

**DOI:** 10.3390/sports12040102

**Published:** 2024-04-02

**Authors:** Chad McLean, Andrew P. Lavender, Ethan Pereira, Kerry Peek, Paul Davey, Fadi Ma’ayah, Susan Morris, Julia Georgieva

**Affiliations:** 1 Curtin School of Allied Health, Curtin University, Bentley, WA 6102, Australias.morris@curtin.edu.au (S.M.); julia.georgieva@curtin.edu.au (J.G.); 2 Institute of Health and Wellbeing, Federation University Australia, Ballarat, VIC 3350, Australia; 3 Discipline of Physiotherapy, Faculty of Health Sciences, University of Sydney, Lidcombe, NSW 2006, Australia; kerry.peek@sydney.edu.au; 4 Curtin School of Nursing, Curtin University, Bentley, WA 6102, Australia; p.davey@curtin.edu.au; 5 School of Education, Curtin University, Bentley, WA 6102, Australia; fadi.maayah@curtin.edu.au

**Keywords:** football, heading, subconcussion, subconcussive head impact

## Abstract

The purpose of this study is to investigate the acute effects of ball pressure on anticipation timing following a series of purposeful headers in adult football (soccer) players. There is evidence to suggest acute neurophysiological changes to the brain following purposeful heading; this may lead to altered anticipation timing as a result, potentially having future safety implications for players. A repeated measures crossover design was used. Seventeen participants aged between 20 and 30 years performed (i) 20 rotational headers with a lower-pressure match ball (58.6 kPa; 8.5 psi), (ii) 20 rotational headers with a higher-pressure match ball (103.4 kPa; 15 psi), or (iii) 20 non-headers (kicks) as a control each on separate days. The effect of ball pressure on anticipation timing accuracy, measured as absolute, constant, and variable errors, was assessed before and immediately after each intervention session using an anticipation timing task. Differences between group means were compared using repeated measures ANOVA and linear mixed effects models, with *p*-values of <0.05 considered statistically significant. No significant differences in anticipation timing accuracy across interventions were detected between control, occluded, and non-occluded trials. This finding differs from the previous literature regarding the measurable, acute effects of purposeful heading. The anticipation timing task may lack sensitivity for detecting the effects of repeated heading on brain function.

## 1. Introduction

Purposeful heading is a skill unique to football, whereby players use any part of their head to intentionally initiate contact with the ball in an attempt to change its trajectory [1]. During a purposeful header, the head is subjected to a degree of acceleration as a result of the recoil magnitude experienced from the impact with the ball [2]. As the ball leaves the head, the skull recoils in one direction, while the brain accelerates in the opposite direction [3,4], which may lead to stress and strain to brain tissue via shearing and rotational forces [3]. Rapid head movement, even in the absence of direct head contact, can lead to deformation of the brain tissue via the same shearing and rotational forces [5]. Although the vast majority of purposeful headers have been shown to be non-injurious [6], Zutrauen et al. [7] observed that approximately 9% of reported head injuries in football were due to purposeful heading [8]. With players experiencing anywhere from 1 to 9 headers per match [9], there are increasing concerns regarding the potential for damage to the brain [10]. Previous studies have investigated the neurophysiological effects of head impacts during various sports in the short term, such as transient periods of reduced cognitive and motor function following 3 min sparring bouts in boxers [11] and following routine tackling drills in rugby players [12]. One of the first studies to investigate the effects of heading on brain function specifically was by Di Virgilio et al. [13], who found that football players demonstrated an acute reduction in corticomotor function following a series of 20 purposeful headers [10]. More recently, a study by Ashton et al. [14] observed significant reductions in performance on the King–Devick test and with measures of working memory following a series of 20 purposeful headers using both soft (60.7 kPa; 8.8 psi) and hard (111.7 kPa; 16.2 psi) balls.

Factors such as the location of impact, the type of header, sex, neck strength and girth, and ball properties (e.g., size, mass, pressure) have been shown to influence head acceleration during purposeful heading [2,3]. While most of these factors are significant and should be considered in heading safety, ball pressure is easily modifiable and remains understudied. A recent study by Peek et al. [15] demonstrated that headers from lower-pressure balls resulted in lower head accelerations during purposeful headers when compared with higher-pressure balls. Specifically, ball pressure may directly influence the recoil force magnitude experienced by the player, subsequently altering acceleration of the head and reducing the risk of injury to the brain with a lower-pressure ball [1]. Currently, the International Football Association Board (IFAB) guidelines explicitly state that competition balls should be within a pressure range of 58.6 kPa (8.5 psi) to 107.56 kPa (15.6 psi) [2,15]. This substantial variance suggests that players may head balls that are at a higher pressure than necessary for play [16] and at a pressure higher than the pressure recommended by individual ball manufacturers. This is particularly important as ball pressure may not be routinely measured accurately with a pressure gauge prior to matches or training, potentially resulting in players experiencing an unnecessarily high impact force to the head.

Another variable that could influence head acceleration during heading is related to individual skill development and anticipation. During purposeful heading, players must visually track the movement of the ball and estimate its arrival at a certain point in order to make contact with the ball using their forehead. The ability to execute this skill is called anticipation timing [17]. The ability to anticipate is not only important for the performance of the header but also potentially has a role to play in injury prevention. There is emerging evidence to suggest that effective anticipation allows for the execution of certain preparatory actions that may reduce head acceleration following a purposeful header [18]. Actions such as pre-activation of the neck musculature prior to a purposeful header have been shown to reduce head acceleration [3,19,20]. Similarly, a study by Narimatsu et al. [21] included the potential protective effects of teeth clenching (masseter activation) in reducing linear head acceleration following a header. The majority of these studies note the importance of accurately anticipating a head impact in order for players to execute these protective responses. As a result, the effective ability to anticipate and accurately time a head movement during a purposeful header may result in lower head acceleration [18], thereby potentially reducing the risk of injury to the brain.

Anticipation timing requires visual-spatial processing and an effective perceptual–motor interface for successful task completion [17,22]. Recently, it has been shown that anticipation timing can be reliably measured through visual evoked potentials and electroencephalography, providing links to the role of neural pathways and brain structure in reaction time, decision making, and anticipation timing [23]. As there is growing evidence to suggest a short-term effect on neurophysiological function following purposeful heading [10,13], we hypothesize that there may be a subsequent loss of ability to accurately anticipate following a series of purposeful headers. Despite this developing literature, the majority of research on anticipation timing does not directly investigate the effects of repetitive head impacts or headers on brain function [18]. Furthermore, even less is known about the effect ball pressure has on this relationship and whether it should be considered in the pursuit of protecting the brain. The importance of anticipation timing and the modifiability of ball pressure in football presents an opportunity to be proactive and identify strategies that will serve to better protect the brain in order for future football guidelines to be better informed to incorporate practical changes to reduce this risk. The aim of this research was to investigate whether ball pressure has any acute effect on anticipation timing following a series of purposeful headers in adult football players. We hypothesized a reduction in anticipation timing performance in the short term following a series of purposeful rotational headers; further, we also hypothesized that a greater reduction in anticipation timing performance would be seen following a series of headers from a higher-pressure ball compared with a lower-pressure ball.

## 2. Materials and Methods

Nineteen adult football players were recruited for this study. Two participants were excluded from participation via the health-screening questionnaire (Qualtrics) for having previous concussions. This resulted in a total of 17 participants (*n* = 3 females, *n* = 14 males; means ± SD: age 22 ± 3.5 years, age range 20–30 years, height 177 ± 7.9 cm, weight 76.2 ± 14 kg, football-playing experience 10.2 ± 5.9 years).

Ethical approval for this study was granted by the Human Research Ethics Committee (HREC) of Curtin University (HRE2021-6044). We recruited adult football players who had played at least one season of football, prior to commencing the study via distribution of printed flyers, social media, and word of mouth at local football clubs/webpages. Participants were excluded if they answered ‘yes’ to any question in the health-screening questionnaire, which included the following: Do you have any neurological conditions? Have you ever had a traumatic neck injury? Have you ever had a brain injury that resulted in a loss of consciousness? Have you had a concussion within the past 12 months? Do you exclusively play as a goalkeeper? (Goalkeepers are unlikely to head the ball during training or games).

Participants were provided with a participant information sheet, and written consent was obtained from each participant prior to study participation. Eligible and consenting participants attended the laboratory at Curtin University on four separate occasions, with a washout period of at least 72 h to allow for any acute effects on brain function to resolve. Participants visited once for a familiarization session and then three separate times for the intervention sessions. All participants were instructed to avoid vigorous exercise and to consume no caffeine or alcohol for at least 24 h prior to testing. An instruction to abstain from heading in the 48 h period before each session was also given. Upon arrival for the familiarization session, anthropometric and demographic data were collected using Qualtrics software (Qualtrics, Provo, UT, USA). Participants were given a practice run of the anticipation timing task (ATT), which is a simple purpose-built program (Python 3.10.0, Anaconda, Newark, DE, USA) used to measure anticipatory timing accuracy. The task involved tapping a 100-Newton-(N)-force sensor with the index finger of their dominant hand when they anticipated that a red rectangle, moving at a constant velocity in one direction, would intercept a stationary black rectangle—displayed on a 22-inch monitor (Bravia LCD Digital Color TV, Sony, Tokyo, Japan). There were two variations of the ATT, occluded and non-occluded trials, that appeared in random order. During the occluded condition, the moving rectangle disappeared at a fixed point through the trial, and participants were required to predict when it would have intercepted had it not disappeared. The non-occluded condition contained no occlusion of the moving rectangle during the trial. Participants were then familiarized with the heading and kicking protocols that they would be completing during the experimental sessions.

During the experimental sessions, participants first completed 60 trials (30 occluded and 30 non-occluded) of the ATT as a pre-intervention measure. They then completed either (i) 20 rotational headers with a lower-pressure match ball (58.6 kPa; 8.5 psi) [15], (ii) 20 rotational headers with a higher-pressure match ball (103.4 kPa; 15.0 psi) [15], or (iii) 20 kicks (103.4 kPa; 15.0 psi; control group) with 30 s between each header/kick over a 10 min period. Next, they completed another 60 trials of the ATT as a post-intervention measure, starting the trials within 10 s of the last header/kick. Each participant completed all three interventions across the three experimental sessions (repeated measures crossover), at one intervention per session. The allocated order of the interventions was randomized. The heading protocol was adapted from that of Di Virgilio et al. [13], which was designed to simulate routine play. A standard size 5 football (400 g; Deploy Football, Taren Point, NSW, Australia) was launched from a ball delivery machine (Ball Launcher, Globaltec Innovation LTD, Brightlingsea, Essex, UK) at a speed of 30 kph—which was checked before each experimental session using a handheld Stalker Sport 2 radar gun (Stalker Sport, Richardson, TX, USA) from a fixed 1 m mark behind the ball delivery machine. Participants were positioned in a 1 × 1 m box that was 6 m away from the ball delivery machine [15,24]. They were instructed to direct their headers or kicks at a goal positioned directly to their left (90 degrees from the oncoming ball), while keeping their feet on the ground (for heading only). Following the testing sessions, participants were instructed to abstain from vigorous activity for the rest of the day.

A sample size of *n* = 16 was calculated based on 80% power in a within-factor repeated measures ANOVA to show an effect size of f = 0.33 (equivalent to partial eta squared = 0.1, e.g., a 10% difference in the outcome between ball pressures) [25]. IBM SPSS Statistics (version 28.0.1.1 (15)) was used for statistical analyses. Data were compared using interactive mixed effects linear regression models using the condition (occluded vs. non-occluded), experimental session (lower-pressure heading vs. higher-pressure heading vs. kicking control), and time (pre-intervention vs. post-intervention), with *p*-values of <0.05 considered statistically significant. We estimated both fixed and random effects of interventions and conditions on the change from the time (pre-intervention to post-intervention) of the primary outcome. Results were reported according to the variation of the anticipation timing and summarized as mean pre–post differences for each intervention in milliseconds (ms), with corresponding 95% confidence intervals. Mean pre–post differences greater than 150 ms or less than −150 ms were deemed as outliers and were excluded from analysis. Anticipation timing accuracy was recorded in sessions/groups of 60 trials (30 occluded, 30 non-occluded).

In addition, repeated measures ANOVAs were used to investigate differences between times (pre–post) within conditions (occluded, non-occluded) separately using three dependent variables—(1) constant error (CE), the mean positive or negative error; (2) absolute error (AE), the mean non-directional error; and (3) variable error (VE), the standard deviation of the CE—and averaging the data points.

## 3. Results

**Figure 1:**
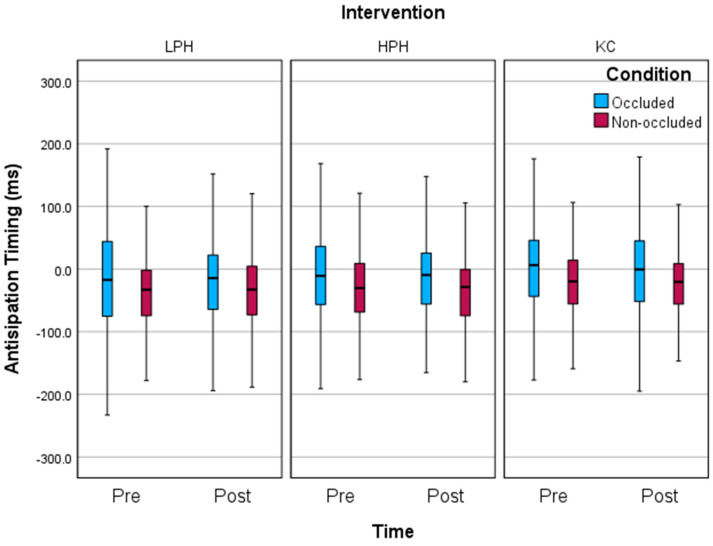
Anticipation timing task (anticipatory timing accuracy) where zero is the target and any negative score indicates timing is early and a positive score indicates timing is late.

Overall, each participant performed 20 headers or kicks per experimental session. One participant’s data were excluded due to data corruption. A total of four mean pre–post difference scores were removed as outliers (two from each ATT variation). Our results showed no significant differences in anticipation timing accuracy using the data from the VE between occluded and non-occluded conditions (Figure 1). The occluded condition was less accurate than the non-occluded condition at both pre- and post-test timepoints, and there was no significant difference in anticipation timing accuracy in the comparison between higher-pressure heading (HPH), lower-pressure heading (LPH), and kicking control (KC) conditions (Table 1).

Statistical analyses of the AE, CE, and VE for the non-occluded and occluded conditions are separately presented in Table 2 and Table 3 next. There were no statistically significant differences detected between the HPH, LPH, and KC for the AE, CE, or VE in the non-occluded and occluded conditions.

## 4. Discussion

In this study, we aimed to determine whether ball pressure impacts anticipation timing accuracy immediately following a series of purposeful headers. Overall, our results showed that neither a higher (103.4 kPa; 15.0 psi) nor a lower (58.6 kPa; 8.5 psi) ball pressure significantly affects anticipation timing accuracy, rejecting our hypothesis.

Our results differ from those of Di Virgilio et al. [13] and Ashton et al. [14], who found acute deficits in the measures of corticomotor and memory function following purposeful heading. However, the study by Di Virgilio et al. did not include a kicking control condition, making it possible that the changes observed in their study were also influenced by factors other than heading alone. One such factor could be slightly higher levels of physical exertion, which has the potential to negatively affect anticipation timing performance [26]. Broadly speaking, our results do not support findings from other sports, such as rugby [12] and boxing [11], and our results showed no significant mean pre–post differences between LPH or HPH compared with KC for both occluded and non-occluded trials for the AE, CE, or VE. Due to the lack of observed differences, our results suggest no short-term measurable effect with anticipation timing of headers with higher- nor lower-pressure balls compared with the kicking control. Potential reasons for the lack of detectable differences across the three conditions might be a lack of sensitivity in the ATT compared with measures such as cortical silence period (cSP) and memory function or the fact that the trials were performed with occluded and non-occluded conditions appearing randomly within the same trial. Furthermore, it is possible that any changes in brain function are more subtle than can be measured using our ATT protocol following an acute bout of heading.

Unsurprisingly, performance on the occluded ATT trials was significantly poorer than that on non-occluded trials irrespective of the intervention, with participants being observed to click the button earlier when they were required to predict the interception of the two rectangles rather than clicking the button while they were able to see it. Contrary to the findings of this study, Ballester et al. [27] reported poorer coincidence anticipation timing performance in normal vision conditions compared with stroboscopic vision conditions. It is suggested that anticipation timing in stroboscopic vision conditions increases visual-spatial memory demands and decreases the loss of attentiveness in participants, resulting in more vigilant participants who produce better anticipation timing performance [27,28]. Furthermore, Ballester et al. [27] also noted that normal vision conditions may be impacted by attentional under-stimulation and monotony, resulting in worse anticipation timing performance. A potential reason for the observed differences in the findings between our study and those of Ballester et al. [27] may be due to the randomization of the occluded and non-occluded ATT trials, which reduced predictability and thereby may have increased attentiveness in both conditions. Furthermore, the duration of the 60 ATT trials was markedly less compared to the coincidence anticipation task used by Ballester et al. [27], reducing the potential effects of attention fatigue in the ATT. Given the significance of occlusion and anticipation in both player performance and safety in football [18], this area remains largely understudied, and our findings may present an avenue for future research.

While our results showed no evidence to support the notion of significant changes in the ATT following heading with low- or higher-pressure footballs, different, or perhaps more sensitive, measures of brain function could produce more detailed findings. Previous studies have produced significant results using transcranial magnetic stimulation (TMS), which has been validated as a reliable measure of corticomotor function [29], measures of balance [12], and memory function [11,13]. In our study, we included a kicking control group to enable a comparison of the effect of heading on the ATT with that of another commonly executed football skill. We recommend that future research consider the inclusion of an additional control group that performs no exercise (no headers or kicks) over the same 10 min period to further reduce any confounding physiological input and produce more vigorous results.

The ball pressures chosen for our study were based on the minimum (58.6 kPa; 8.5 psi) and near-maximum (103.4 kPa; 15.0 psi) extremes of the IFAB-regulated range [2]. While our results demonstrated no significant difference in anticipation timing accuracy between these two pressures, lower-pressure balls can have other advantages. For example, low ball pressures have been shown to increase player control of the ball [30], which may lead to improved performance and subsequent long-term engagement with the sport, particularly in young, developing players. Additionally, the previous literature has shown evidence for reducing head recoil and acceleration with lower-pressure balls [15]. As ball pressure can be easily adjusted before training and matches, perhaps playing with a low ball pressure would add to both the quality and the safety of the sport.

A strength of our study was that we ensured strong standardization between sessions through the use of the same equipment throughout the study, the use of a ball launcher to ensure a constant ball velocity for each header, and a controlled environment for the ATT to eliminate distraction. Further, our heading protocol replicated that of another study [13], which was strengthened through the inclusion of a kicking control condition. Participants were also blind to the allocation of the intervention to reduce bias. We do acknowledge some limitations associated with this study. Our conclusions could have been different if we had used other measures to correlate with the findings from the ATT. The use of TMS to assess the cSP has been shown to be sensitive and provides good support for changes in cortical function following repeated minor head impacts [11,12,13]. Supporting the data from the ATT with cSP measures would strengthen studies assessing the impact of repeated headers on brain function. It would also be interesting to conduct more tests after the intervention at 1 or 2 h to learn more about the recovery period, also known to take at least 1 h but not as long as 24 h [13]. Perhaps reassessing participants using the Rivermead Questionnaire would have provided some insight to participants’ subjective experience of performing the 20 rotational headers.

The study by Di Virgilio et al. [13] included the measures of cognitive function, cSP, and postural control in an attempt to address multiple areas of neurophysiological function. While this provides a more comprehensive overview of any changes in brain function, the equipment used was highly clinical and expensive, making it less accessible to football clubs, particularly at the community level. We chose the ATT as our primary measure as it is a cheaper, more practical, and accessible piece of equipment to measure neurophysiological function. While our study did not demonstrate differences in anticipation timing accuracy across interventions between control, occluded, and non-occluded trials, it is also possible that any changes in brain function may take many years to become observable. For example, we may have observed different results if we had compared a more heterogeneous group of players, including comparing players with a high volume of headers with those with a low volume of accumulative headers across the course of their playing careers. Lastly, our study included fewer females than males, limiting generalizability to a female playing population.

## 5. Conclusions

This is the first study to investigate the relationship between ball pressure and anticipation timing accuracy following an acute bout of heading (along with a kicking control intervention) in adult football players, with our results demonstrating no significant relationship between the assessed conditions. However, the difference we found in our occluded and non-occluded ATT trials irrespective of intervention may provide avenues for further research using alternative, more sensitive outcome measures.

### Practical Implications

Ball pressure does not acutely affect anticipation timing accuracy after a series of purposeful headers in adult football (soccer) players, with no difference in anticipation timing observed between the heading and the kicking control conditions.

Despite limited evidence of significant changes in our pre-post ATT trials, significant differences were observed when comparing occluded versus non-occluded, suggesting that future research on occluded versus non-occluded measures of anticipation timing accuracy is warranted.

The results from this study can be added to the evidence base about the safety implications of heading.

## Figures and Tables

**Table 1:** Mixed effects linear regression analysis of the effect of the intervention conditions on the attention timing task.

Variables	Anticipatory Timing (95% CI)	*p*-Value
**Fixed Effects**		
Grand mean	−2.226 (−107.923, 103, 470)	0.967
Interventions		
KC	Reference	
LPH	−12.701 (−162.126, 136.725)	0.868
HPH	−9.074 (−158.499, 140.352)	0.905
Conditions		
Non-occluded	Reference	
Occluded	−18.455 (−22.391, −14.519)	<0.001
Time		
Pre-intervention	Reference	
Postintervention	−1.534 (−5.470, 2.402)	0.445
**Random Effects**		
SD (intercept)	53.870	
SD (residual)	76.190	
**Model Fit**		
−2Log-likelihood	66,244.67	<0.001

Note: ICC = 41.4% (i.e., 41.4% of the variance in the ATT explained by level 2 variables (intervention, condition, and time).

**Table 2:** Comparison of non-occluded condition ATT trials using ANOVA.

Outcome Measure	Intervention	Mean Pre–Post Difference (ms)	*p*-Value	95% Confidence Interval (ms)	
				Lower Bound	Upper Bound
Absolute error	HPH	−28.4	0.537	−94.1	37.4
	LPH	2.9		−6.1	12.0
	KC	16.2		−16.3	48.7
Constant error	HPH	11.6	0.844	−24.9	48.1
	LPH	2.9		−9.0	14.8
	KC	11.4		−39.2	16.4
Variable error	HPH	−53.1	0.101	−201.7	95.5
	LPH	2.5		−10.3	15.2
	KC	70.0		−68.6	208.6

**Table 3:** Comparison of occluded condition ATT trials using ANOVA.

Outcome Measure	Intervention	Mean Pre–Post Difference (ms)	*p*-Value	95% Confidence Interval (ms)	
				Lower Bound	Upper Bound
Absolute error	HPH	−56.9	0.459	−168.7	54.8
	LPH	−18.2		−38.0	1.6
	KC	9.0		−2.0	20.0
Constant error	HPH	40.9	0.743	−43.8	125.6
	LPH	2.3		−21.2	25.9
	KC	−8.7		−22.3	4.9
Variable error	HPH	−105.7	0.222	−307.6	96.2
	LPH	50.2		−125.1	24.6
	KC	24.8		−2.2	51.8

## Data Availability

The data that support the findings of this study are available from the corresponding author (A.P.L.) upon reasonable request.
